# Reduced Brain Activity in the Right Putamen as an Early Predictor for Treatment Response in Drug-Naive, First-Episode Schizophrenia

**DOI:** 10.3389/fpsyt.2019.00741

**Published:** 2019-10-08

**Authors:** Renrong Wu, Yangpan Ou, Feng Liu, Jindong Chen, Huabing Li, Jingping Zhao, Wenbin Guo, Xiaoduo Fan

**Affiliations:** ^1^Department of Psychiatry, The Second Xiangya Hospital of Central South University, Changsha, China; ^2^National Clinical Research Center for Mental Disorders, Changsha, China; ^3^Department of Radiology, Tianjin Medical University General Hospital, Tianjin, China; ^4^Department of Radiology, The Second Xiangya Hospital of Central South University, Changsha, China; ^5^University of Massachusetts Medical School, UMass Memorial Medical Center, One Biotech, Worcester, MA, United States

**Keywords:** schizophrenia, early predictor, olanzapine, fractional amplitude of low frequency fluctuation, pattern classification

## Abstract

Antipsychotic medications can have a significant effect on brain function after only several days of treatment. It is unclear whether such an acute effect can serve as an early predictor for treatment response in schizophrenia. Thirty-two patients with drug-naive, first-episode schizophrenia and 32 healthy controls underwent resting-state functional magnetic resonance imaging. Patients were treated with olanzapine and were scanned at baseline and 1 week of treatment. Healthy controls were scanned once at baseline. Symptom severity was assessed within the patient group using the Positive and Negative Syndrome Scale (PANSS) at three time points (baseline, 1 week of treatment, and 8 weeks of treatment). The fractional amplitude of low frequency fluctuation (fALFF) and support vector regression (SVR) methods were used to analyze the data. Compared with the control group, the patient group showed increased levels of fALFF in the bilateral putamen at baseline. After 1week of olanzapine treatment, the patient group showed decreased levels of fALFF in the right putamen relative to those at baseline. The SVR analysis found a significantly positive relationship between the reduction in fALFF after 1 week of treatment and the improvement in positive symptoms after 8 weeks of treatment (*r* = 0.431, *p* = 0.014). The present study provides evidence that early reduction and normalization of fALFF in the right putamen may serve as a predictor for treatment response in patients with schizophrenia.

## Introduction

Antipsychotic medications are the standard treatment for schizophrenia. However, up to 30% of patients with schizophrenia respond poorly to current antipsychotic treatment (1). Therefore, it is of great clinical value if we can develop a biomarker either before the treatment or during the early treatment course to predict clinical outcome. However, previous effort trying to explore clinically useful predictors has mostly failed (2, 3, 4).

Recently, functional magnetic resonance imaging (fMRI) provides insights into the underlying pathophysiological mechanisms of schizophrenia and antipsychotic effects on brain function. Antipsychotic medications are dopamine receptor antagonists or partial agonists (5). Antipsychotic treatment modulates intrinsic brain activity in the cortical (i.e., the prefrontal cortex and anterior cingulate cortex) and subcortical regions (i.e., the basal ganglia and amygdala) in patients with schizophrenia, which constitute the thalamocortical network (6, 7, 8, 9). Results from longitudinal investigations indicate a normalization and a concurrent denormalization of intrinsic brain activity related to antipsychotic treatment in schizophrenia (2, 3, 4). We have reported that olanzapine increases functional connectivity (FC) in the default-mode network (DMN) and sensorimotor circuits and decreases FC in the left superior temporal gyrus (10, 11, 12) in schizophrenia. In addition, Lui et al. observed a significant relationship between improvement in clinical symptoms and alterations of brain activity in drug-naive, first-episode schizophrenia (13). Another study suggested that prefrontal lobe dysfunction predicts treatment response in drug-naive, first-episode schizophrenia (14).

Although the abovementioned studies provide useful information for us to understand antipsychotic treatment effect on brain activity in schizophrenia, several important issues in the existing literature need to be addressed. First, in most previous studies, patients were exposed to different antipsychotic medications. For example, risperidone, olanzapine, clozapine, aripiprazole, and sulpiride were used in a study with first-episode schizophrenia (13). Different antipsychotic medications may have differential effects on brain function as reported in positron emission tomography (PET) studies (15, 16). Second, chronic patients with a long illness duration were recruited in some previous studies (10, 11, 12). The illness duration itself may have a neurotoxic effect on brain gray matter (17). Third, many previous studies have focused on specified brain regions or networks using a region-of-interest (ROI) method (18, 19). It is not surprising that different studies reported different findings when different ROIs were used. Although the findings are meaningful, the ROI method may not include all significant brain regions that are related to the core pathological alterations in schizophrenia.

Taking these potentially important confounding factors into consideration, the present study was to examine whether acute changes in the brain, 1 week after olanzapine treatment, can predict clinical response after 8 weeks of olanzapine treatment in drug-naive, first-episode schizophrenia because antipsychotic drugs may have acute effects on brain function after only 1 week of treatment when it is in general still too early to observe meaningful clinical improvement (20). Fractional amplitude of low frequency fluctuation (fALFF) was used to analyze the imaging data in a voxel-wise and whole-brain fashion. Support vector regression (SVR) analysis, one of pattern classification approaches, was used to predict individual level clinical response after 8 weeks of olanzapine treatment.

## Methods

### Participants

A total of 68 right-handed participants were recruited, including 34 patients with drug-naive, first-episode schizophrenia and 34 healthy controls. Patients were recruited from the Department of Psychiatry, the Second Xiangya Hospital of Central South University in China, and healthy controls were recruited from the local community. Schizophrenia was diagnosed using the Structural Clinical Interview for DSM-IV (SCID), patient version (21). All participants had more than 6 years of formal education and aged from 18 to 50 years old. All participants had routine laboratory tests and a physical exam, and met the following exclusion criteria: any psychiatric disorders other than schizophrenia and any medical disorders. For healthy controls, those who have a first-degree relative with psychiatric disorders were excluded.

The study was approved by the ethics committee of the Second Xiangya Hospital of Central South University, China. Each participant provided a written informed consent to participate in the study.

### Procedure

Clinical symptoms were assessed using the Positive and Negative Syndrome Scale (PANSS), which includes the positive symptoms, negative symptoms, and general psychopathology subscales (22). All patients were treated with olanzapine as clinically appropriate and tolerated (week 1: dose range 10–20 mg/day with mean and standard deviation 12.19 and 3.09, respectively; week 8: dose range 10–30 mg/day with mean and standard deviation 18.59 and 4.96, respectively). For patients, clinical symptom assessment and the imaging scan were performed twice (pre-treatment baseline, after 8 weeks of treatment). For healthy controls, the imaging scan was performed at baseline.

### Data Acquisition and Preprocessing

MRI images were obtained using a 3T MRI scanner (Siemens Verio, Erlangen, Germany). The participants were required to remain motionless and awake with their eyes closed. Soft earplugs and foam pads were used to decrease scanner noise and head motion. Resting-state fMRI images were obtained with a gradient-echo echo-planar imaging (EPI) sequence using the following parameters: repetition time/echo time = 2,000 ms/30 ms, 33 slices, 64 × 64 matrix, 90° flip angle, 22 cm field of view, 4 mm slice thickness, no slice gap, and 240 volumes (480 s).

The images were preprocessed in Matlab (R2012b) using the DPABI software (V4.1_190725) (23) and SPM8. After slice timing and head motion correction, participants with over 2 mm maximal translation and 2° maximal rotation were excluded. Several covariates, including Friston-24 head motion parameters (6 head motion parameters, 6 head motion parameters one time point before, and 12 corresponding squared items) acquired *via* rigid body correction (24), signal from a ventricular region of interest, and signal from a region centered in the white matter, were removed. The global signal was not removed as it is still a controversial practice in the resting-state fMRI field (25). The data were then normalized to conventional EPI template in the Montreal Neurological Institute (MNI) space and resampled to 3 × 3 × 3 mm^3^ voxels. Finally, the images were bandpass-filtered (0.01–0.08 Hz) and linearly detrended.

### Calculation of fALFF

The analysis procedure for fALFF was performed according to the methods described in a previous study (26). First, the time course of each voxel was converted to the frequency domain without bandpass filtering with a fast Fourier Transform (FFT) and the power spectrum was acquired. Then, the square root was calculated at each frequency of the power spectrum because the power of a given frequency was proportional to the square of the amplitude of its frequency component, and the averaged square root was obtained across 0.01–0.08 Hz at each voxel. The sum of amplitude across 0.01–0.08 Hz was divided by that across the whole frequency range. Finally, the fALFF of each voxel was divided by the global mean fALFF value within a brain mask for standardization purpose (27). This method has been well applied in psychiatric disorders, such as schizophrenia (28, 29, 30), major depressive disorder (27, 31, 32), somatization disorder (33), and healthy subjects (34).

### Statistical Analyses

Demographic and clinical data were analyzed using chi-square test and two-sample *t*-test as appropriate.

Group comparisons between patients and healthy controls were performed at baseline to identify brain regions with abnormal values of fALFF in schizophrenia. For those abnormal brain regions, paired-sample *t*-test was performed within the patient group to compare values of fALFF between 1 week after olanzapine treatment and baseline. Framewise displacement (FD) was calculated for each participant according to the method described in a previous study (35). The mean FD, age, gender, years of education, and individual dose of olanzapine were used as covariates of no interest. The significance level of *p* value was corrected for multiple comparisons based on the Gaussian random field (GRF) theory (voxel significance: *p* < 0.001, cluster significance: *p* < 0.05) using the software REST (36).

### Improvement in Clinical Symptoms

We calculated the reduction rate (RR) of the PANSS total scores using the following formula.

$${\text{RR}}_{\text{total}\_8\text{w}}\cdot =\cdot \left({\text{PANSS}}_{\mathrm{total}\_0}\cdot -\cdot {\text{PANSS}}_{\mathrm{total}\_8\text{w}}\right)\cdot /\cdot {\text{PANSS}}_{\text{total}\_0}$$

Note: RR_total_8w_ refers to the reduction rate of the PANSS total score after 8 weeks of treatment. PANSS_total_0_ is the PANSS total score at baseline, whereas PANSS_total_8w_ is the PANSS total score after 8 weeks of treatment. The RR of each PANSS subscale was calculated using a similar formula.

### Pattern Classification

Pattern classification was conducted using the LIBSVM software (http://www.csie.ntu.edu.tw/∼cjlin/libsvm/), including a set of machine learning-based algorithms to differentiate two or more groups based on high-dimensional data such as functional images. In the present study, an SVR was performed using a “leave-one-out” procedure and based on the mean fALFF values of the identified cluster. The purpose of SVR was to examine whether changes of fALFF after 1 week of olanzapine treatment can predict improvement in clinical symptoms after 8 weeks of treatment as reflected by the RRs of the PANSS total and subscale scores.

## Results

### Characteristics of the Participants

**Figure 1:**
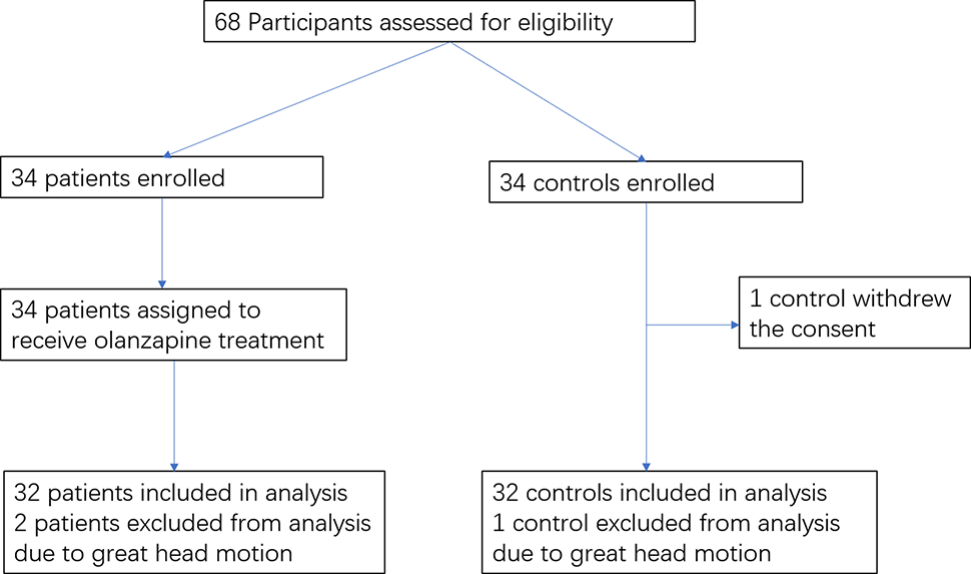
Flowchart of the study participants.

The flowchart of the study participants is shown in **Figure 1**. The imaging scans from three participants (two patients and one healthy control) were discarded due to excessive head motion; one healthy control withdrew the consent. The final analysis included 32 patients and 32 healthy controls. There were no significant differences between the two groups in gender, age, years of education, and the mean FD value (*p*’s > 0.20) (**Table 1**).

**Table 1:** Characteristics of the study participants at baseline.

	Patients (n = 32)	Controls (n = 32)	*p* value
Gender (male/female)	16/16	21/11	0.21
Age (years)	30.94 ± 8.25	31.37 ± 7.84	0.83
Years of education (years)	12.13 ± 3.19	12.03 ± 2.38	0.89
FD (mm)	0.03 ± 0.02	0.03 ± 0.02	0.92
Illness duration (months)	8.91 ± 6.39		
Dose of olanzapine at week 1 (mg/day)	12.19 ± 3.09		
Dose of olanzapine at week 8 (mg/day)	18.59 ± 4.96		

FD, framewise displacement.

### Improvement in Clinical Symptoms Within the Patient Group After 8 Weeks of Treatment

At baseline, the PANSS total score, positive symptoms score, negative symptoms score, and general psychopathology score were 77.38 ± 5.17/20.00 ± 4.32/20.59 ± 3.46/36.78 ± 3.68, respectively (mean ± SD). A significant reduction was found on the PANSS total and subscale scores after 8 weeks of treatment compared to baseline (*p*’s < 0.001) (**Table 2**).

**Table 2:** The PANSS scores within the patient group.

PANSS	Baseline	week 1	week 8
	Mean ± SD	Mean ± SD (*p* value)	Mean ± SD (*p* value)
Positive symptoms scores	20.00 ± 4.32	18.81 ± 4.76 (*p* = 0.217)	8.91 ± 1.55 (*p* < 0.001)
Negative symptoms scores	20.59 ± 3.46	20.56 ± 3.45 (*p* = 0.967)	8.97 ± 1.84 (*p* < 0.001)
General psychopathology subscale scores	36.78 ± 3.68	35.13 ± 3.63 (*p* = 0.035)	18.63 ± 1.48 (*p* < 0.001)
Total scores	77.38 ± 5.17	74.50 ± 5.61 (*p* = 0.017)	36.50 ± 2.97 (*p* < 0.001)

PANSS, Positive and Negative Syndrome Scale; p values represent the comparisons of the PANSS scores between week 1 or week 8 and baseline.

### Baseline Differences in fALFF Between Patients and Healthy Controls

**Figure 2:**
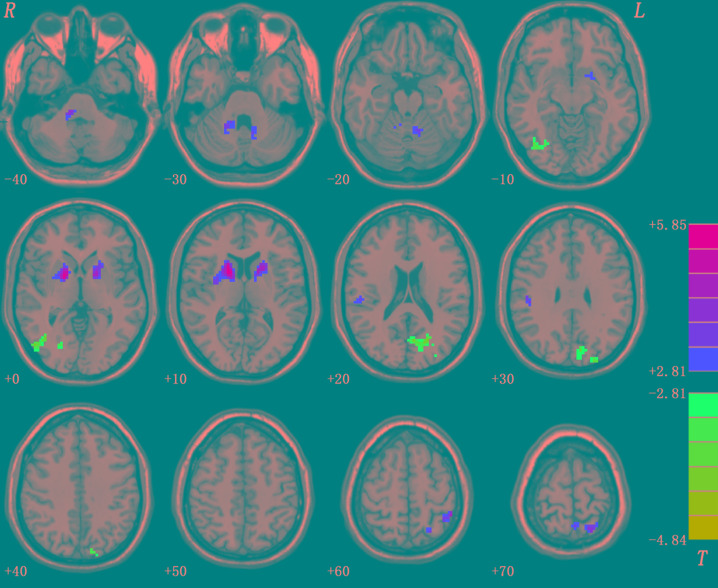
Baseline differences in fALFF between patients and healthy controls. The figure is presented using the viewer of the REST software. Color bars indicate *t*-values of the group analysis. Red color denotes increased fALFF in the patient group compared to the control group, whereas blue color denotes decreased fALFF in the patient group compared to the control group (*p* < 0.05, corrected by the GRF method). fALFF, fractional amplitude of low frequency fluctuation; GRF, Gaussian random field.

At baseline, compared with the control group, the patient group showed increased levels of fALFF in the bilateral putamen/caudate/pallidum, right cerebellum pons, left cerebellum culmen, right inferior parietal lobule, left inferior parietal lobule, and left superior parietal lobule, and decreased levels of fALFF in the left medial frontal gyrus (orbital part), right fusiform gyrus/lingual gyrus, left calcarine cortex/precuneus, and left superior occipital lobe (**Figure 2** and **Table 3**).

**Table 3:** Baseline differences in fALFF between patients and healthy controls.

Cluster location	Peak (MNI)			Number of voxels	*T* value
	x	y	z		
Bilateral Putamen/Caudate/Pallidum	15	3	12	403	5.8517
Right Cerebellum Pons	12	-36	-39	83	4.0648
Left Cerebellum Culmen	-12	-54	-24	41	3.7255
Right Inferior Parietal Lobule	48	-27	33	32	4.5249
Left Inferior Parietal Lobule	-48	-45	60	36	4.3778
Left Superior Parietal Lobule	-18	-60	72	70	4.2737
Right Fusiform Gyrus/Lingual Gyrus	27	-69	-6	115	-4.2794
Left Calcarine Cortex/Precuneus/Superior Occipital Lobe	-6	-69	18	174	-4.8369

MNI, Montreal Neurological Institute; fALFF, fractional amplitude of low frequency fluctuation.

### Alterations in fALFF After 1 Week of Olanzapine Treatment Within the Patient Group

**Figure 3:**
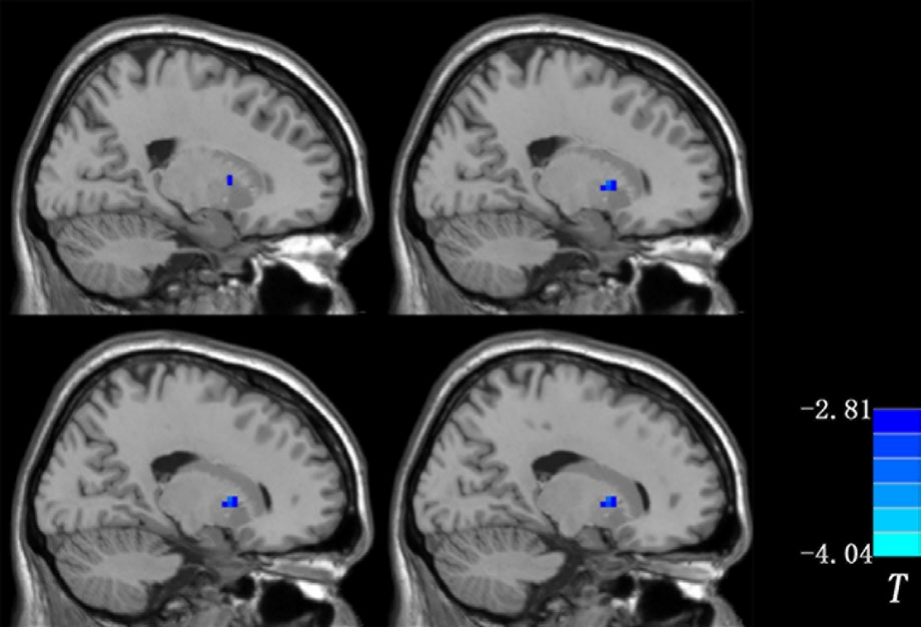
Changes in fALFF after 1 week of olanzapine treatment within the patient group (the right putamen). The comparisons were limited to the brain regions with abnormal levels of fALFF at baseline. The figure is presented using the viewer of the REST software. Color bar indicates *t*-values of the group analysis. Blue color denotes decreased fALFF after 1 week of olanzapine treatment relative to the values at baseline (*p* < 0.05, corrected by the GRF method). fALFF, fractional amplitude of low frequency fluctuation; GRF, Gaussian random field.

After 1 week of treatment, the patient group showed decreased and normalized levels of fALFF in the right putamen relative to those at baseline (**Figure 3** and **Table 4**). For other brain areas with abnormal levels of fALFF at baseline, no significant changes were found after 1 week of olanzapine treatment within the patient group.

**Table 4:** Treatment effect on fALFF after 1 week of olanzapine treatment within the patient group.

Cluster location	Peak (MNI)			Number of voxels	*T* value
	x	y	z		
Right Putamen	15	12	3	22	-3.7892

MNI, Montreal Neurological Institute; fALFF, fractional amplitude of low frequency fluctuation.

### Pattern Classification Results

**Figure 4:**
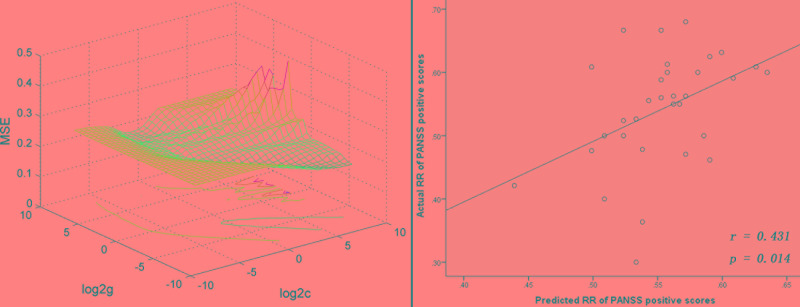
SVR analysis indicates that decreased levels of fALFF in the right putamen after 1 week of olanzapine treatment may predict improvement in positive symptoms after 8 weeks of olanzapine treatment. Left: 3D visualization of SVR results with the best parameters (best c = 22.6274, g = 0.015625) using the grid search method. Right: A positive correlation between predicted and actual reduction rates of individual level PANSS positive symptoms subscale scores after 8 weeks of treatment (*r* = 0.431, *p* = 0.014). The predicted reduction rates were calculated based on decreased levels of fALFF in the right putamen after 1 week of olanzapine treatment using the LIBSVM software. SVR, support vector regression; MSE, mean square error; fALFF, fractional amplitude of low frequency fluctuation; PANSS, the Positive and Negative Syndrome Scale.

Since the right putamen was the only brain region that showed decreased and normalized fALFF after 1 week of olanzapine treatment, the change in levels of fALFF in the right putamen at week 1 might be able to predict treatment response at week 8. To test this possibility, SVR analysis was conducted. The SVR result showed a significantly positive relationship between decreased levels of fALFF at week 1 and the improvement in positive symptoms at week 8 as measured by the RR of the PANSS positive symptoms subscale scores (*r* = 0.431, *p* = 0.014, **Figure 4**).

## Discussion

To our knowledge, the present study was the first to explore an early imaging biomarker to predict antipsychotic treatment response in drug-naive, first-episode schizophrenia using a combination of fMRI data and pattern classification methods. We found that reduced and normalized fALFF in the right putamen after 1 week of olanzapine treatment may serve as a predictor for improvement in positive symptoms after 8 weeks of olanzapine treatment.

Our findings are in line with a previous report (37), which observed increased levels of ALFF in the right putamen in patients with or without auditory hallucinations in first episode schizophrenia. Our findings are also consistent with the results from another study that showed that medicated patients with schizophrenia had decreased metabolic rates in the putamen relative to never-medicated patients (38). Further, our findings are consistent with a previous study that reported that increased gray matter (GM) volume in the right putamen might be a marker to predict antipsychotic treatment response in schizophrenia (39). In that study, patients with drug-naive, first-episode schizophrenia received antipsychotic treatment for 6 weeks. The imaging data were analyzed using a tensor-based morphometry method. It was found that increased GM volume in the right putamen after 6 weeks of treatment was associated with improvement in positive symptoms of schizophrenia. Together with these studies, the present study suggests that structural and functional changes in putamen in response to antipsychotic treatment might be associated with clinical outcome in patients with schizophrenia.

The putamen, a subcortical node of the striatum, is rich in dopamine, a main neurotransmitter in the brain. Dopamine plays a key role in complex behavior and cognition in schizophrenia (40). According to the dopamine hypothesis in schizophrenia, excessive dopamine in the striatum (including the putamen) is associated with positive symptoms such as hallucinations and delusions (41). Antipsychotic medications target multiple dopaminergic receptors including D1 and D2 in the striatum, which might contribute to improvement in positive symptoms. Therefore, reduced and normalized fALFF in the right putamen, even in the early treatment course, was associated with improvement in positive symptoms in the present study.

Our study has several novel aspects. First, we identified a possible imaging biomarker at 1 week of treatment to predict clinical response at 8 weeks of treatment. Clinically, it usually takes 6–8 weeks to determine clinical effectiveness of antipsychotic treatment. Therefore, establishing an early predictor for treatment response is of great clinical value to help psychiatrists decide, during the early treatment course, whether the patient should stay on the chosen antipsychotic medication or switch to a different one. Second, a ROI method was commonly used in previous studies; this method focuses on selected brain regions but may miss other brain regions that are critical to the underlying pathophysiology of schizophrenia (19). In contrast, the fALFF analysis, which takes a voxel-wise whole-brain approach, was used in our study. Third, most previous studies that tried to identify predictors for treatment response used univariate statistics, which is appropriate for group level prediction (42); in contrast, SVR analysis, a pattern classification technique used in our study, is a promising tool for individual level prediction (43). Fourth, the present study included patients with drug-naive, first-episode schizophrenia, minimizing several important possible confounding factors, such as a long illness duration and prior exposure to antipsychotic medication.

The mean age of patients seems older than in typical first-episode patients, such as patients in our previous study (44). Different inclusion criteria on age were used in our two studies. The previous study was conducted in Guangxi and included participants aged from 16 to 30 years, whereas the present study was performed in Changsha and included participants aged from 18 to 50 years. The typical age range for first episode psychosis is 16–35 years old. In the present study, most patients fell into this age range and only four patients were older than 35 years. Hence, the patients in the present study in general still represent usual first-episode drug-naive patients with schizophrenia.

The present study has several limitations. First, the relatively small sample size may limit the generalizability of the results from the present study. Second, all patients were treated with olanzapine; the findings from this study may not be able to generalize to other antipsychotic medications. Third, imaging scans were not done 8 weeks after treatment. Therefore, it is unclear whether the changes in fALFF observed at week 1 persisted at week 8. Future studies with a larger sample size are needed to further examine the clinical utility of using acute changes in fALFF in the right putamen to predict antipsychotic treatment response in patients with schizophrenia.

In conclusion, the present study provides evidence that early reduction and normalization of fALFF in the right putamen may serve as a predictor for treatment response in patients with schizophrenia.

## Data Availability Statement

All datasets generated for this study are included in the manuscript/supplementary files.

## Ethics Statement

The study was approved by the ethics committee of the Second Xiangya Hospital of Central South University, China. Each subject provided a written informed consent to participate in the study. The patients/participants provided their written informed consent to participate in this study.

## Author Contributions

Study concept and design: WG, XF, and JZ. Acquisition, analysis, or interpretation of data: FL, JC, HL, YO, and RW. Drafting of the manuscript: RW, WG, and XF. Critical revision of the manuscript for important intellectual content: WG, XF, and FL. Statistical analysis: WG and FL. Obtained funding: WG and JZ. Administrative, technical, or material support: WG, FL, JC, HL, RW, and JZ. Study supervision: WG and JZ.

## Funding

This study was supported by grants from the National Key R&D Program of China (Grant Nos. 2016YFC1307100 and 2016YFC1306900), the National Natural Science Foundation of China (Grant Nos. 81571310, 81771447, and 81630033), and the Natural Science Foundation of Tianjin (Grant No. 18JCQNJC10900).

## Conflict of Interest

XF has received research support or honoraria from Alkermes, Neurocrine, Avanir, Allergen, Otsuka, Lundbeck, Boehringer Ingelheim, Merck, and Janssen.

The remaining authors declare that the research was conducted in the absence of any commercial or financial relationships that could be construed as a potential conflict of interest.
